# Mechanical and hydrodynamic effects of stent expansion in tapered coronary vessels

**DOI:** 10.1007/s10237-022-01605-1

**Published:** 2022-07-22

**Authors:** Xiangkun Liu, Wen Zhang, Ping Ye, Qiyi Luo, Zhaohua Chang

**Affiliations:** School of Health Science and Engineering, University of Shanghai for Science and Technology, 516 Jungong Road, Shanghai, 200093 China

**Keywords:** Tapered coronary vessels, Equal diameter expansion, Conical expansion, Finite element analysis, Computational fluid dynamics

## Abstract

Percutaneous coronary intervention (PCI) has become the primary treatment for patients with coronary heart disease because of its minimally invasive nature and high efficiency. Anatomical studies have shown that most coronary vessels gradually shrink, and the vessels gradually become thinner from the proximal to the distal end. In this paper, the effects of different stent expansion methods on the mechanical and hemodynamic behaviors of coronary vessels and stents were studied. To perform a structural-mechanical analysis of stent implantation, the coronary vessels with branching vessels and the coronary vessels with large bending curvature are selected. The two characteristic structures are implanted in equal diameter expansion mode and conical expansion mode, and the stress and mechanical behaviors of the coronary vessels and stents are analyzed. The results of the structural-mechanical analysis showed that the mechanical behaviors and fatigue performance of the cobalt-chromium alloy stent were good, and the different expansion modes of the stent had little effect on the fatigue performance of the stent. However, the equal diameter expansion mode increased distal coronary artery stress and the risk of vascular injury. The computational fluid dynamics analysis results showed that different stent expansion methods had varied effects on coronary vessel hemodynamics and that the wall shear stress distribution of conical stent expansion is more uniform compared with equal diameter expansion. Additionally, the vortex phenomenon is not apparent, the blood flow velocity is slightly increased, the hydrodynamic environment is more reasonable, and the risk of coronary artery injury is reduced.

## Introduction

The incidence and mortality rates of coronary heart disease caused by cardiovascular stenosis are increasing, and they are much higher compared with those of most other diseases, which is a serious global health concern (Larsen 2011). Efficient stent intervention with minimal invasion has gradually become the gold-standard treatment for coronary heart disease (Khan et al. 2012), and stent performance plays a vital role in the therapeutic outcome. Different stent structures provide different mechanical behaviors to the stents, such as expansion uniformity, compliance, and fatigue strength. Implantation damage crucially depends on these mechanical behaviors. Therefore, research on the mechanical behaviors of stents is critical. The mechanical behavior of the stent is closely related to vascular injury, and it is widely acknowledged that the vascular stress caused by stent expansion, deformation of the stent and vessel, and intimal hyperplasia induced by hemodynamic changes after stent implantation are the leading causes of intravascular restenosis (Morishita et al. 1997; Spyridopoulos et al. 1997).

Stent implantation has mainly been studied in equal section circular, straight vessels. Finite element analysis (FEA) has been employed to simulate the mechanical behaviors of stents in the expansion process, as well as the expansion uniformity, radial resilience, and axial shortening of stents with different geometric structures (Chua et al. 2003; Shen et al. 2014). Stents with varied structures will have different forms of deformation in the round and straight vessels, and the stress on the vascular wall will also be different. High stress is expected at both ends of the stents; however, anatomical assessments show that most blood vessels gradually become thinner and shrink from the proximal to the distal end (Ragkousis et al. 2015). Timmins et al. (2008) studied the effects of different stent behaviors on the expansion uniformity in tapered vessels. When stents expand in gradually shrinking vessels, they demonstrate different adhesion characteristics because of the uneven stress of stents. Stent flexibility is essential for providing support capacity, and it reflects stent consistency with vascular movement after implantation. Some postoperative adverse reactions are also closely related to adhesion between the stent and vessel. If the stent has good flexibility, it fits perfectly in the blood vessel after implantation, which reduces blood vessel damage. Petrini et al. (2004) established two different support models to simulate and analyze the influence of support structures on compliance and self-contact. When self-contact occurs, the bending stiffness of the support increases sharply.

Intravascular restenosis is related to structural mechanics and the state of blood flow after stent implantation. The forms of the stent after implantation have an impact on blood flow. The affected blood flow will cause local eddy currents of blood in the blood vessel, reduce blood flow, and lead to kinetic energy loss. The normal physiological activities of vascular wall cells may be hindered. The stent strut will hinder the laminar flow of blood and create a disturbance. Two low-pressure areas are formed in front of and behind the strut. Eddy currents generated in these two areas (especially in serious cases) will cause the blood to pool. The blood flow velocity above the strut is higher than the normal level, resulting in considerable shear stress and platelet activation. Platelet aggregation to a critical concentration in the two low-pressure areas will eventually lead to a coagulation cascade. Simultaneously, endothelialization of the vascular surface in the low shear stress area is inhibited, thus promoting restenosis (Jimenez and Davies 2009).

In the study of coronary artery hemodynamics, it is difficult to distinguish subtle fluid changes only through clinical observation. Stone et al. (2012) combined and statistically analyzed results from many clinical studies, but such analyses can only deduce the treatment effect, making it difficult to directly determine the blood flow with high accuracy. Therefore, computational fluid dynamics (CFD) can be used as an auxiliary method to analyze the implanted blood vessels more systematically and intuitively. In recent years, simulations of stents in various three-dimensional flow fields have become popular (Chen et al. 2017; Hsiao et al. 2014). Gundert et al. (2012), Simao et al. (2017), and Koskinas et al. (2012) have analyzed the blood flow in blood vessels after stent implantation and studied its impact on the blood flow environment focusing on different vascular morphologies and stent diameters. Martina Bukac et al. (2019) simulated four contact forms between stents and blood vessels, mainly focusing on the degree of fit between the stent and blood vessel under different bending situations and the influence of small-scale eddy currents around the stent strut on the wall. Lotfi and Barber (2014) analyzed the case in which the implanted stent’s diameter exceeded the artery’s diameter and compared the difference in local shear stress under conditions of different diameters. Poon et al. (2014) conducted in-depth research on T-shaped stents, which may cause chaotic flow at the intersection of blood vessels. The flow field of a simplified model is simulated by the CFD method to study the influence of wall shear stress and hemodynamics in the bifurcation region. Yu et al. (2017) studied the implantation of tapered stents. Different expansion forms during stent implantation significantly impact hemodynamics because of the large taper of real coronary vessels. The impact on blood flow morphology after the implantation of an ideal round and straight intravascular stent has been studied. However, the impact after the implantation of a curved conical intravascular stent in the physiological environment requires further evaluation.

This study analyzed the mechanical behaviors of stents implanted with tapered vessels using the finite element method. The behaviors of different forms of stent vascular systems were analyzed, focusing on the stress and strain of the stents and vessels. The equal diameter expansion mode and conical expansion mode in the conical vessel were compared. Stent recovery under the vessel’s action and the stress of the stent and the vessel were also studied. There have been many studies on FEA and CFD of coronary stents, but there has been little research on FEA and CFD of equal diameter expansion mode and conical expansion mode of coronary stents in tapered vessels. In the analysis of compliance performance, the stent expands in the curved conical vessel by equal diameter expansion mode and conical expansion mode. The balloon expands the stent during the expansion and assumes a round and straight morphology in the curved vessel. After the expansion, the balloon shrinks, and the stent conforms freely to the curvature of the vessel. The mechanical behaviors of the stent and vessel with different expansion methods were compared. Then, the characteristics of vascular hemodynamics in each system, including blood velocity, wall shear stress, and pressure distribution, were studied by computational fluid dynamics. In this study, we proposed a new balloon-expandable stent method. This study assessed the influence of different stent expansion methods on the stent and vascular performance, providing a theoretical basis for product development and clinical research and promoting the growth of the coronary stent industry and future clinical development.

## Methods

### Reconstruction of a three-dimensional model of coronary artery

**Fig. 1:**
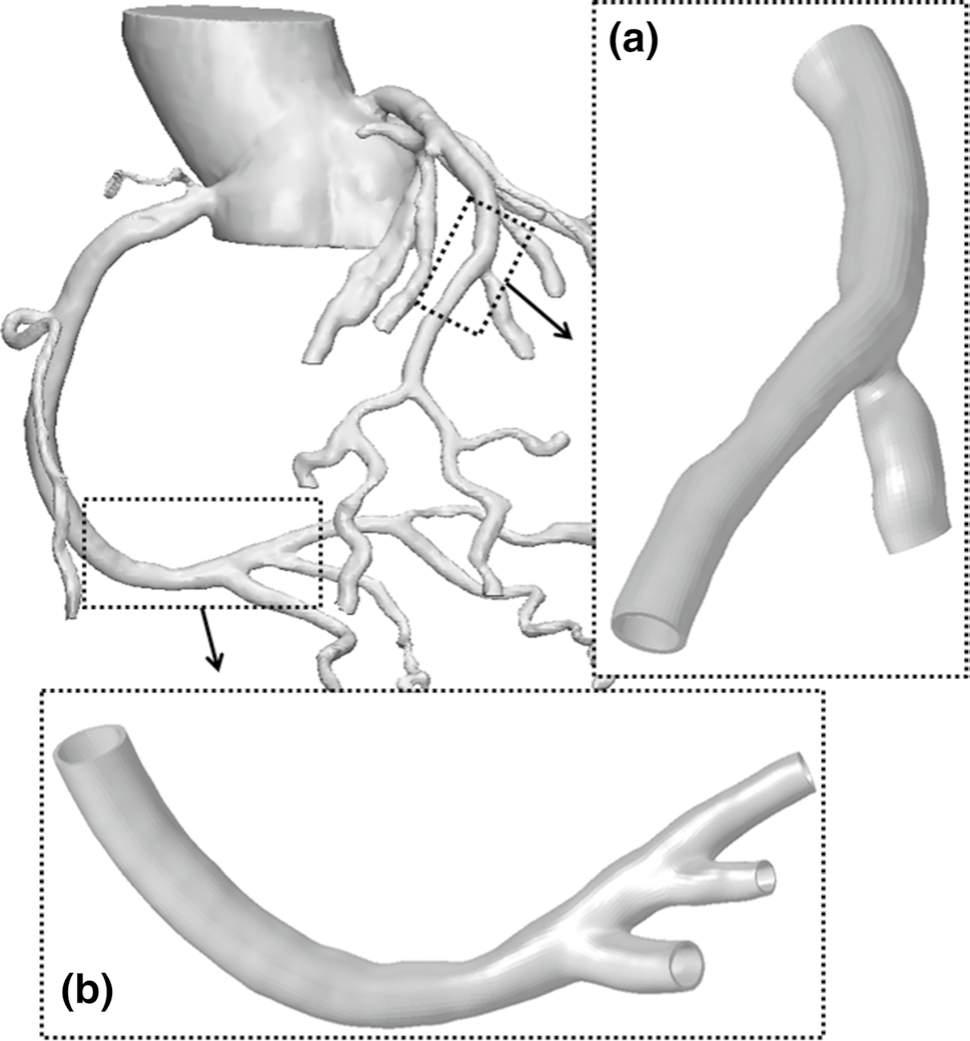
Reconstruct model of coronary vessels: **a** branch coronary vessels and **b** curved coronary vessels

The coronary artery model used in this study was reconstructed from a healthy male volunteer’s computed tomography angiography (CTA) data. The data were imported into Mimics (Materialise Corp., Leuven, Belgium) in DICOM format, and the coronary blood basin model was extracted according to the gray image value and density threshold. After the image data were imported, the upper, lower, front, and rear spatial orientations are determined, and the software automatically calculates and generates the axial, coronal, and sagittal views according to the original scanned image. The threshold analysis curve was generated by locating the target area, and the threshold range value was set (with all corresponding areas were selected). The selected area contained a hot pixel (such as the non-vascular areas with similar behaviors) which was filtered out using the growth function in Mimics. Finally, the rough three-dimensional shape of the vascular tissue structure is reconstructed through calculation. After smoothing and optimization, a vascular geometric model was derived for subsequent simulation analysis and calculation. This study analyzed two kinds of coronary vessel morphology, one is the coronary vessel with an expanded position at the branch vessel, and the other is the more curved and tapered coronary vessel. Figure 1 shows these two kinds of vessel morphology. The discrete vascular model uses the c3d8h element, which can accurately simulate the incompressible material of blood vessels. The mechanical behavior of the blood vessel was modeled through a homogeneous, isotropic, and hyperelastic constitutive model introduced by Holzapfel et al. (2005). Constitutive law was based on a sixth-order reduced polynomial strain energy density function as

1 $$S={C}_{10}({\overline{I} }_{1}-3)+{C}_{20}({\overline{I} }_{1}-3{)}^{2}+{C}_{30}({\overline{I} }_{1}-3{)}^{3}+{C}_{40}({\overline{I} }_{1}-3{)}^{4}+{C}_{50}({\overline{I} }_{1}-3{)}^{5}+{C}_{60}({\overline{I} }_{1}-3{)}^{6}$$

2 $${\overline{I} }_{1}={\overline{\lambda }}_{1}^{2}+{\overline{\lambda }}_{2}^{2}+{\overline{\lambda }}_{3}^{2}$$

3 $${\overline{\lambda }}_{i}={{J}^{-}}^\frac{1}{3}{\lambda }_{i}$$

where ${\overline{I} }_{1}$ is the first deviatoric strain invariant, ${\overline{\lambda }}_{i}$ and ${\lambda }_{i}$ are deviatoric and principal stretches, respectively, and *J* is the total volume ratio. Material parameters are summarized in Table 1 (obtained from (Azaouzi et al. 2012)).

**Table 1:** Material coefficients of the blood vessel

Coefficient	*C*_10_	*C*_20_	*C*_30_	*C*_40_	*C*_50_	*C*_60_
Value	0.00652	0.0489	0.00926	0.76	-0.43	0.0869

### Finite element analysis of the coronary stent intervention

**Fig. 2:**
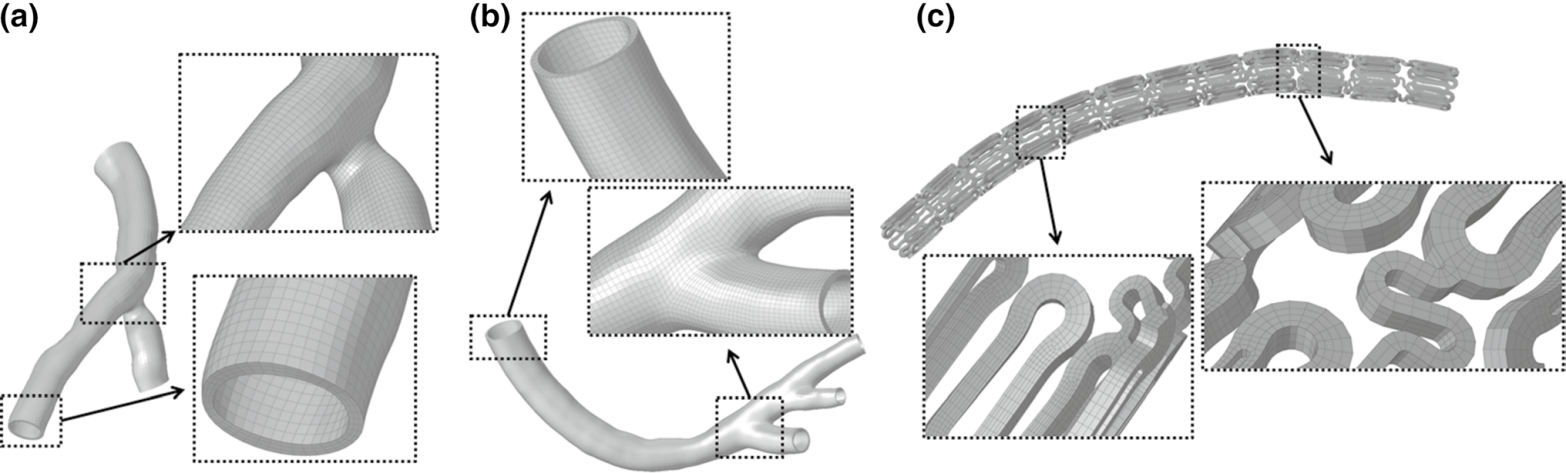
**a** Branch coronary vessels mesh model, **b** curved coronary vessels mesh model and **c** stent mesh model

Coronary stents are used to treat ischemic heart disease. The stent is sent to the vascular stenosis area by the delivery system and expanded using a balloon to support the diseased vessels. The finite element analysis of stent implantation refers to both the stent production and processing and the clinical intervention processes. The stent crimping analysis involved the crimping of the stent to the surface of the folded balloon, and then the combination was delivered to the conical personalized blood vessel through the conveyor. The balloon was then filled and expanded until the stent was completely occupied by the balloon. Finally, the balloon was withdrawn to complete the stent implantation. The coronary stent selected in this study was Firehawk® (MicroPort Medical (Group) Cop., Shanghai, China) coronary stent. According to the size information of the vascular model, two stent types were selected to complete the finite element analysis of stent implantation. The outer diameter of the stent is 1.8 mm, and the wall thickness is 0.086 mm. The three-dimensional model of the support was established using SolidWorks (SolidWorks Corp., Concord, MA, USA). The finite element analysis simulation method in Abaqus/Explicit (Dassault Systemes Simulia Corp., Providence, RI, USA) was applied to the analysis, solution, and post-processing for stent implantation. The stent is made of an L605 cobalt-chromium alloy pipe, with an elastic modulus of 220 GPa, Poisson’s ratio of 0.3, yield stress of 515 MPa, and tensile strength of 1400 MPa. The C3D8I element in hexahedral nonconforming mode was selected for the finite element model, and the mesh division of the vessels and stent is shown in Fig. 2. The coronary artery balloon is made of polymeric material with a thickness of 0.025 mm. The material used for analysis was simplified into an isotropic linear elastic material model with an elastic modulus of 890 MPa and a Poisson’s ratio of 0.4 (Umer et al. 2019). The balloon model is a reduced integral membrane unit M3D4R.

**Fig. 3:**
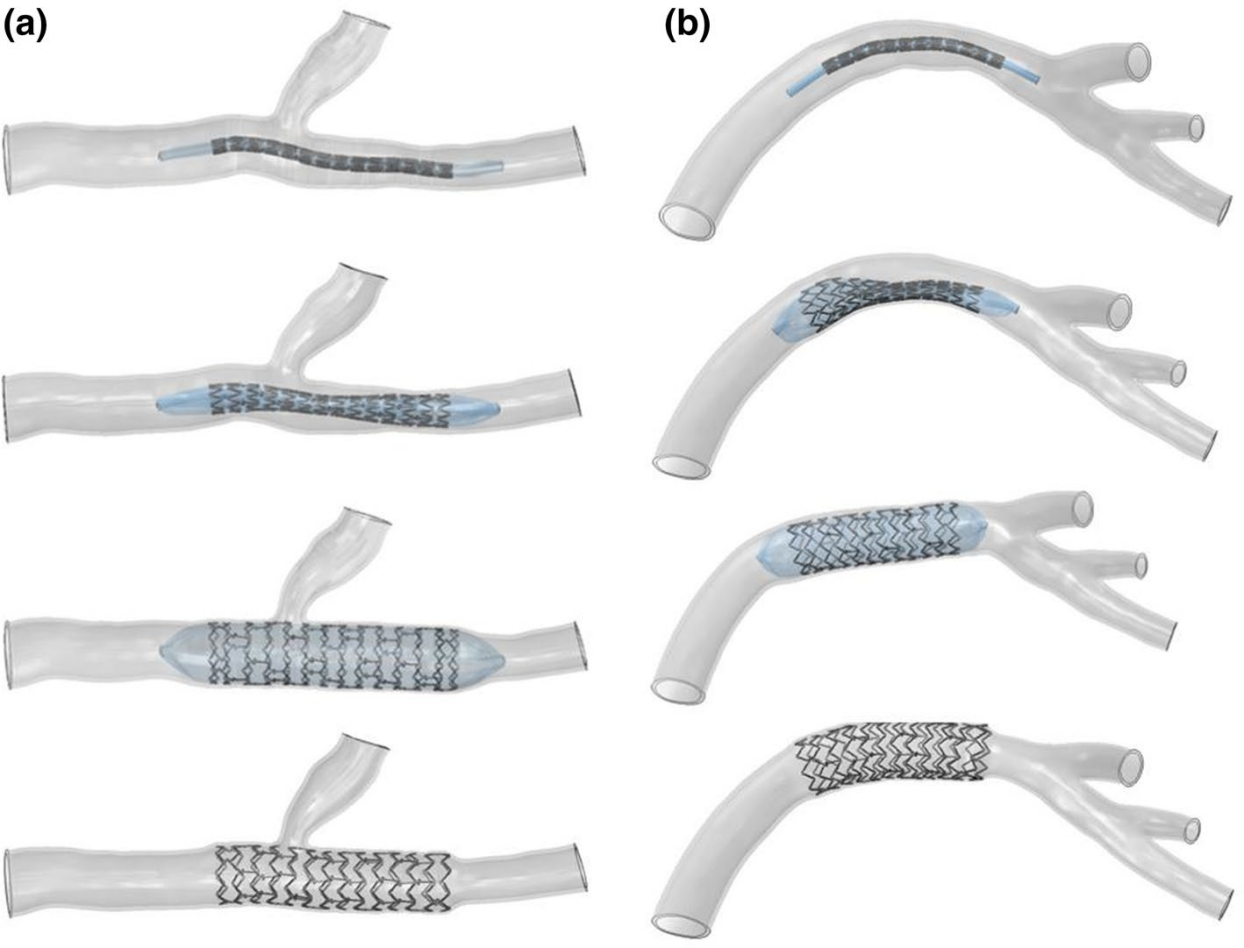
The process of stent implantation: **a** stent implantation with branch coronary vessels and **b** stent implantation with curved coronary vessels

The crimping simulation uses a cylindrical rigid tube to crimp the stent to the balloon surface instead of the crimping tool. The contact between the support and the crimping tool adopts a point to surface contact, the outer surface of the support is the secondary surface, and the inner surface of the crimping tool is the main surface. Point to surface contact is also adopted between the stent and the balloon, with the inner surface of the stent as the primary surface and the outer surface of the balloon as the secondary surface. In the definition of contact behaviors, the penalty function method of nonlinear penalty stiffness is used for normal contact performance. A displacement load to the tool tube was applied to squeeze the stent from the original size to the required size of the support. The delivery analysis was used to deliver the stent and balloon as a whole to the designated vascular position. By applying displacement boundary conditions to the front end of the crimping tool, the stent and balloon are transported to the specified position as a whole. Then the crimping tool is removed, and the stent rebounds freely to simulate the squeezing and rebound process. Stent expansion analysis is used to apply pressure on the inner surface of the balloon, and balloon expansion drives stent expansion until the stent completely fits within the blood vessel. In this analysis, the contact between the stent and the blood vessel adopts a point-to-surface contact, with the outer surface of the stent as the primary surface and the inner surface of the blood vessel as the secondary surface. Finally, the balloon was removed, and the finite element analysis of stent intervention was completed. To study different intervention methods according to the characteristics of conical vessels, the balloons used in stent expansion are equal diameter and conical balloons, respectively. The process of stent implantation of coronary vessels is shown in Fig. 3.

The proximal diameters of coronary vessels at the two locations are about 3.272 mm and 4.274 mm, respectively, and the distal diameters are about 2.696 mm and 3.200 mm, respectively, showing an apparent conical state. Different stent specifications were selected according to the size of the blood vessels. The first selection had a stent specification with an expansion size of 3.5 mm, and the latter selection had a stent specification with an expansion size of 4.5 mm. For these two vascular models, four different balloon models were designed: two equal diameter balloons and two conical balloons. The balloon expansion parameters are shown in Table 2.

**Table 2:** The balloon expansion parameters (mm)

Vessel model	Expansion mode	Proximal diameter	Distal diameter
Branch coronary vessels	Equal diameter expansion	3.665	3.665
	Conical expansion	3.665	3.020
Curved coronary vessels	Equal diameter expansion	4.787	4.787
	Conical expansion	4.787	3.584

### Computational fluid dynamics method

After the finite element analysis of the coronary post-stent implantation, a hemodynamic analysis was carried out using ANSYS CFX (ANSYS Inc., Canonsburg, PA, USA). The primary purpose of the fluid analysis is to study the effects of different forms of blood vessels and different expansion methods on their hemodynamics after stent implantation. In hydrodynamics calculations, the shape of the blood area after stent model implantation was extracted, and the stent and blood vessel wall were reset. The discrete solid model of fluid analysis was established based on the extracted model. Then the meshing size was adjusted to 0.15 mm, at which point the simulation cost and the model resolution were calculated. To obtain more accurate wall-related parameters, five boundary layers were established during meshing. The number of radial expansion model units of branch coronary vessels was 2,737,092, the number of nodes was 804,588, the number of conical expansion model units of branch coronary vessels was 2,758,636, the number of nodes was 806,696, the number of equal diameter expansion model units of curved coronary vessels was 2,444,422, the number of nodes was 713,077, the number of conical expansion model units of curved coronary vessels was 2,429,282, and the number of nodes was 706,266. In this study, to make the inlet conditions consistent with the actual blood flow state, a fluid extension area was added at the inlet end, which made the blood flow fully divergent. When the blood flow entered the real inlet area of the coronary artery, the divergence was the distribution of velocity varying with the section radius.

**Fig. 4:**
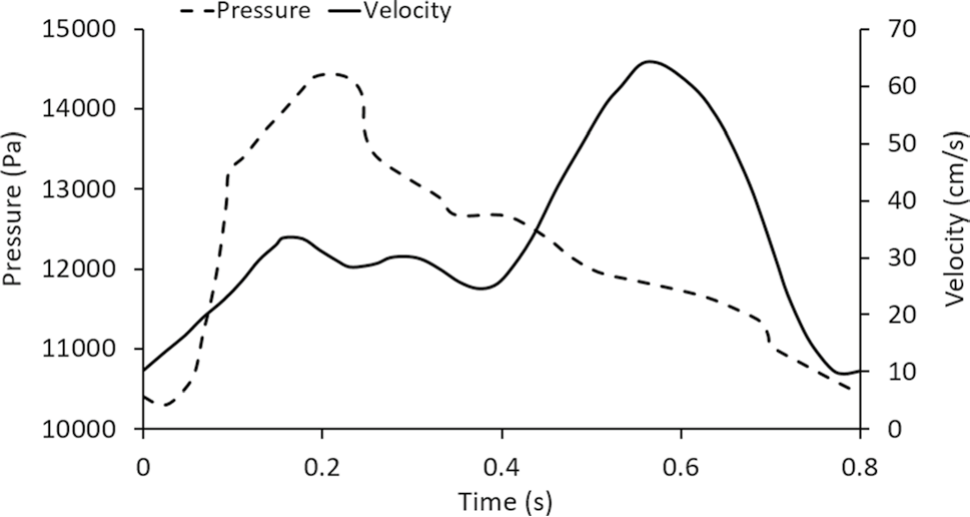
The velocity inlet and pressure outlet conditions under the pulsating periodic load

The boundary conditions of fluid analysis referred to the literature data of Martina Bukac et al. (2019) and selected the velocity inlet and pressure outlet conditions under the pulsating periodic load (Fig. 4). To better reflect the stability of the calculation, this study calculated two pulsation cycles and took the analysis results of the corresponding time for post-processing. In fluid analysis, blood is considered an incompressible Newtonian fluid. The laminar blood model and blood flow pulsation were loaded for transient flow analysis. The blood density is 1050 kg/m^3^, and the viscosity is 0.0035 Pa·S. To analyze the blood flow state in the blood vessel, the Reynolds number in the simulation is less than 2000, and the flow state is laminar. Since the diameter of blood vessels is greater than 1 mm, blood can be regarded as Newtonian fluid (Aenis et al. 1997). The intravascular flow satisfies the laws of mass and momentum conservation, as evidenced by the continuity and Navier–Stokes equations.

4 $$\frac{\partial {u}_{k}}{\partial {x}_{k}}=0$$

$\rho (\frac{\partial {u}_{j}}{\partial t}+{u}_{k}\frac{\partial {u}_{j}}{\partial {x}_{k}})=-\frac{\partial p}{\partial {x}_{j}}+\eta (\frac{{\partial }^{2}{u}_{k}}{\partial {x}_{k}^{2}}$) (5). where $u$ is velocity, $t$ is time, $x$ is a coordinate tensor, $P$ is pressure, $\uprho$ is density, and $\upeta$ is the apparent viscosity term.

## Results

### The effects of different expansion methods on the mechanical behaviors of stents and vessels

According to the finite element analysis of stent implantation, the “dog bone phenomenon” occurred at both ends during stent expansion, which is a common phenomenon of coronary balloon expansion stents. During the process of equal diameter expansion and conical expansion, the tilting trend of the stent was relatively consistent, and it did not affect the dog bone phenomenon because of the different shapes of the balloon. The shape of the coronary artery curved coronary vessels was relatively curved, and the expansion speed at both ends of the stent differed during the expansion process because there was no synchronous expansion. This was primarily because the stent expansion was related to the initial bending shape. Conversely, the balloon length was too long, and the uneven pressure during filling led to this phenomenon.

**Fig. 5:**
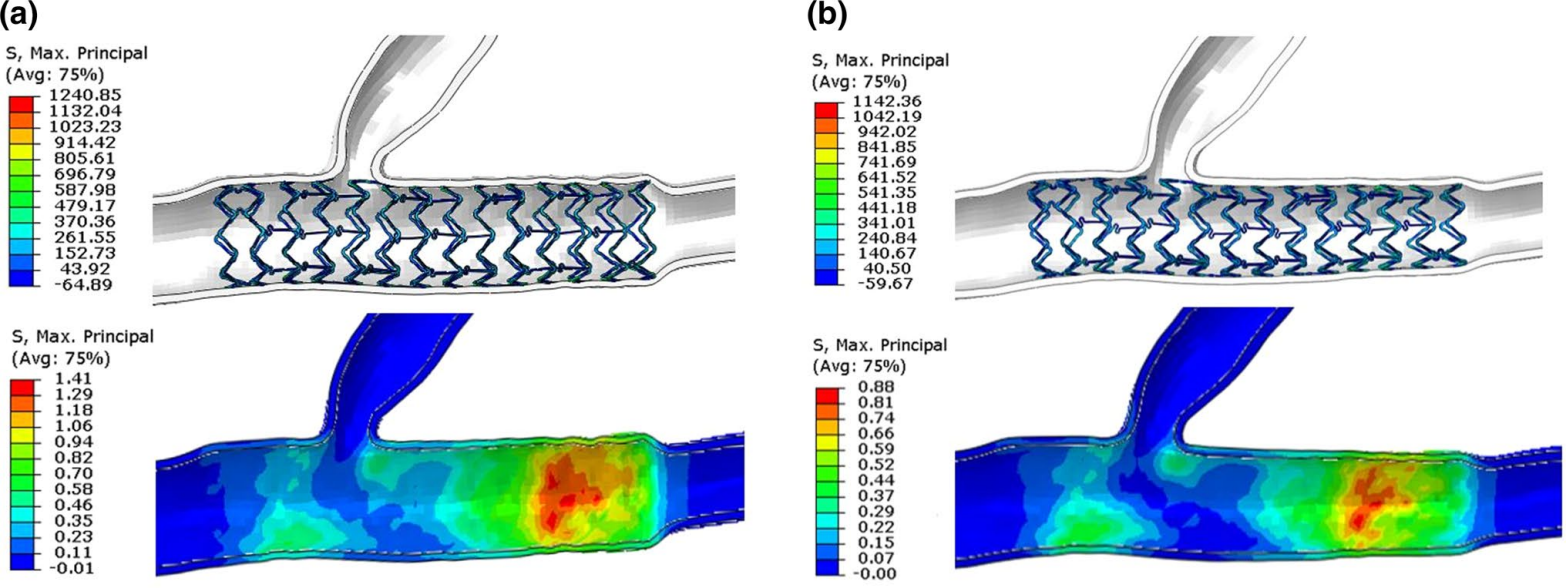
Finite element analysis results of branch coronary vessels: **a** equal diameter expansion and **b** conical expansion

**Fig. 6:**
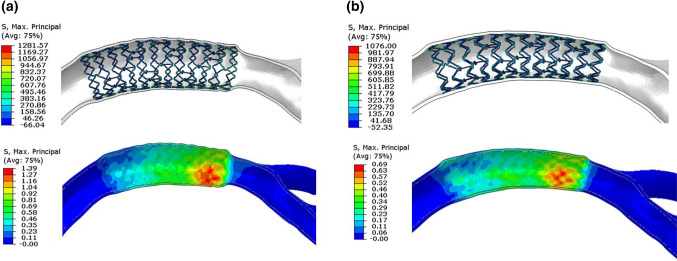
Finite element analysis results of curved coronary vessels: **a** equal diameter expansion and **b** conical expansion

The stent material used for this study was the L605 cobalt-base alloy metal material. A fracture and fatigue analysis of the material was performed based on stress analysis. Exceeding the maximum tensile stress of the stent was the leading cause of material fracture. Under the influence of different stent expansion methods, the mechanical behavior of vascular damage was also determined by the maximum principal stress analysis. The maximum principal stress of the stent after implantation was 1076.0–1281.6 MPa. This stress value varied by about 200 MPa in different vascular forms and expansion modes. There were significant differences in the maximum principal stress of blood vessels. In branch coronary vessels, the maximum principal stress of blood vessels in equal diameter expansion mode was 1.41 MPa (Fig. 5a), and that in conical expansion was 0.88 MPa (Fig. 5b). The damage to blood vessels caused by equal diameter expansion was much greater than that of conical expansion. In curved coronary vessels, the stress of blood vessels in equal diameter expansion mode was 1.39 MPa (Fig. 6a), and the conical expansion was 0.69 MPa (Fig. 6b). Figure 5 shows the finite element analysis results of branch coronary vessels under equal diameter expansion and conical expansion. Figure 6 shows the finite element analysis results of curved coronary vessels under equal diameter expansion and conical expansion. The analysis results showed that the vascular stress concentration areas caused by the two expansion methods were primarily distributed in the vessels with a smaller diameter. It can be seen that the conical expansion mode has an impact on the stress distribution, and the stress distribution at the contact between the blood vessel and the stent was more uniform. In the equal diameter expansion mode, the stress on the blood vessel was insignificant at the location with a large diameter. With the decrease in diameter, the higher stress area noticeably increased, and the blood vessel produced a large deformation. The volume of the stent implantation location of branch coronary vessels and curved coronary vessels was 44.5 mm^3^ and 63.0 mm^3^, respectively. Table 3 compares the volume of blood vessels with a maximum principal stress greater than 0.8 MPa or greater than 0.5 MPa at the stent implantation location. A significant finding is that the high-stress area in blood vessels resulting from equal diameter expansion had a significantly higher magnitude compared with that caused by conical expansion (Table 3). This study shows that the effects of different expansion modes on blood vessels are very different and can be influenced by changing the expansion mode.

**Table 3:** The volume of different stress areas at the stent implantation location of blood vessels

Vessel model	Expansion mode	> 0.8 MPa	> 0.5 MPa
Branch coronary Vessels	Equal diameter expansion	6.148 mm^3^	14.383 mm^3^
	conical expansion	0.076 mm^3^	4.957 mm^3^
Curved coronary vessels	Equal diameter expansion	8.460 mm^3^	27.844 mm^3^
	Conical expansion	0.000 mm^3^	1.523 mm^3^

### The effects of different expansion methods on hemodynamics

**Fig. 7:**
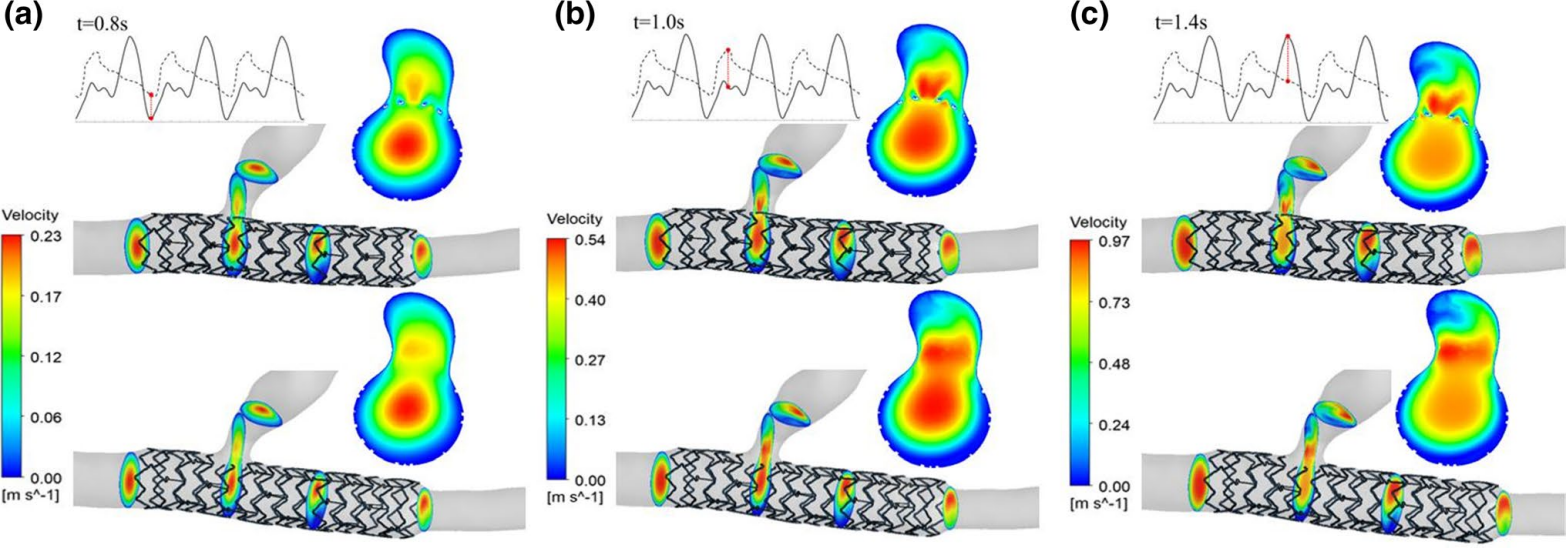
The velocity distribution of the branch coronary vessels in the equal diameter expansion at specific time points before and after windowing: **a** 0.8 s, **b** 1.0 s and **c** 1.4 s

**Fig. 8:**
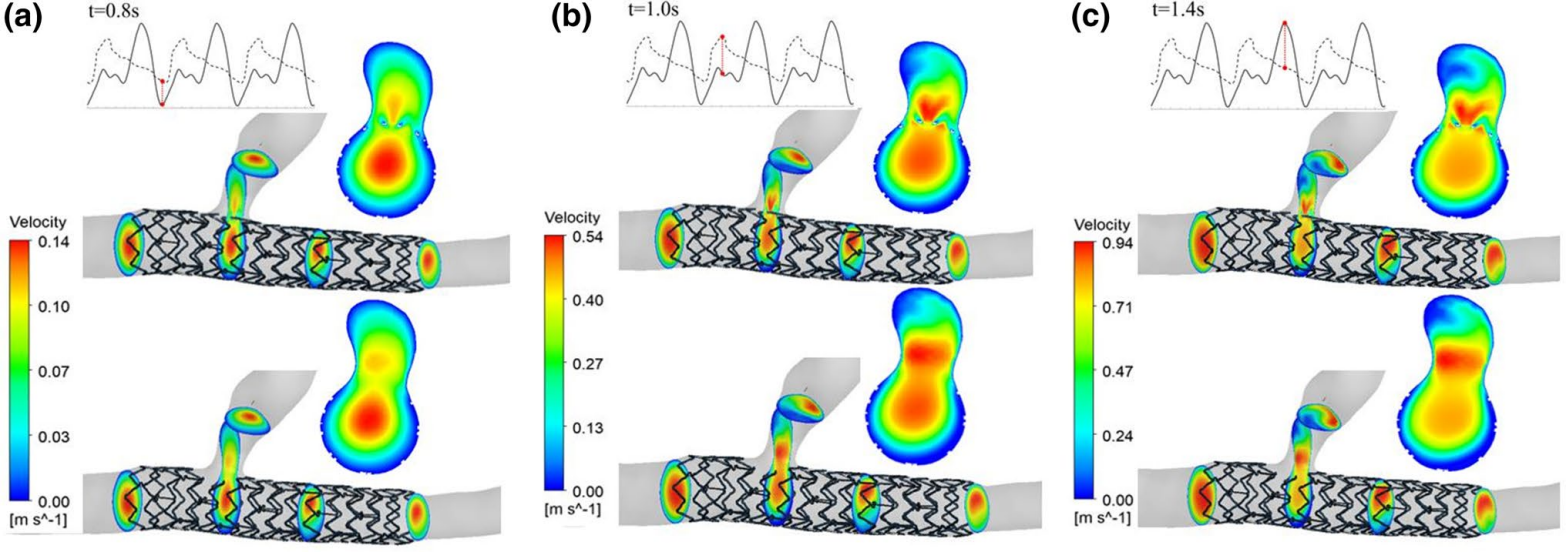
The velocity distribution of the branch coronary vessels in the conical expansion at specific time points before and after windowing: **a** 0.8 s, **b** 1.0 s and **c** 1.4 s

**Fig. 9:**
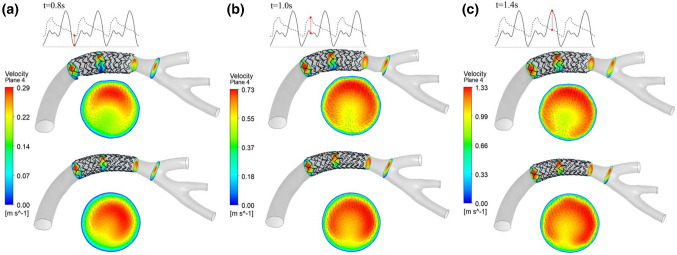
The velocity distribution of the curved coronary vessels in equal diameter expansion and conical expansion at specific time points: **a** 0.8 s, **b** 1.0 s and **c** 1.4 s

The different expansion modes of stents in coronary vessels affect the vessel morphology and blood flow. This study analyzed the effects of different forms of vessels and their expansion modes on blood flow. For branch coronary vessels, there is a branch vessel at the stent implantation position, and some structures of the stent are at the blood flow inlet of the branch vessel. Different treatment methods of the stent wave rod also impact the blood flow at the bifurcation. The hemodynamic analysis model of this study established the complete stent implantation model and the stent windowing model at the branch vessel. The windowing model is an approximate treatment to delete the stent wave rod at the branch vessel, and the hydrodynamics of these two models were studied, respectively. After completing an analysis of the two pulsation periods, the results of the velocity distribution along the axial section at specific time points (0.8, 1.0, and 1.4 s) are selected. Figure 7 shows the velocity distribution of the branch coronary vessels in the equal diameter expansion at specific time points before and after windowing. Figure 8 shows the velocity distribution of the branch coronary vessels in the conical expansion at specific time points before and after windowing. Previous studies have shown that the blood flow velocity distribution in the branch coronary vessels is slightly different in each region and that the maximum axial section velocity gradually increases from the entrance. Because of the influence of branch vessels, the high-speed area on the distal section of the vessel is not in the center but tends to the side with branches. The different stent treatments at the branch make the blood flow velocity distribution at the bifurcation significantly different. For the untreated stent, there is reduced flow velocity in the high-speed area of blood flow, a risk of blood stasis, and the stent wave rod structure results in local blood flow obstruction. Conversely, the stent with fenestration has little effect on the blood flow of branch vessels. In the clinic, the effect of stent implantation on branch vessels can be reduced by stent fenestration. Figure 9 shows the velocity distribution of the curved coronary vessels in equal diameter expansion and conical expansion at specific time points. It can be seen that the maximum axial cross-sectional velocity is located at the periphery of the section where the curvature radius of the vessel is large. The six fluid analysis models produce blood flow disorder and eddy currents to varying degrees. This study shows that the vortex phenomenon at the end of the equal diameter expansion stent is more evident than conical expansion stent. The existence of the vortex promotes the adhesion and aggregation of blood cells, making the substances in the blood stay in the vortex area for a longer time, thus increasing the risk of thrombosis.

**Fig. 10:**
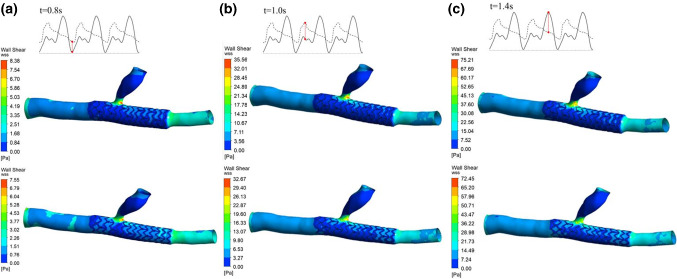
The wall shear stress distribution of the branch coronary vessels in equal diameter expansion and conical expansion at specific time points: **a** 0.8 s, **b** 1.0 s and **c** 1.4 s

**Fig. 11:**
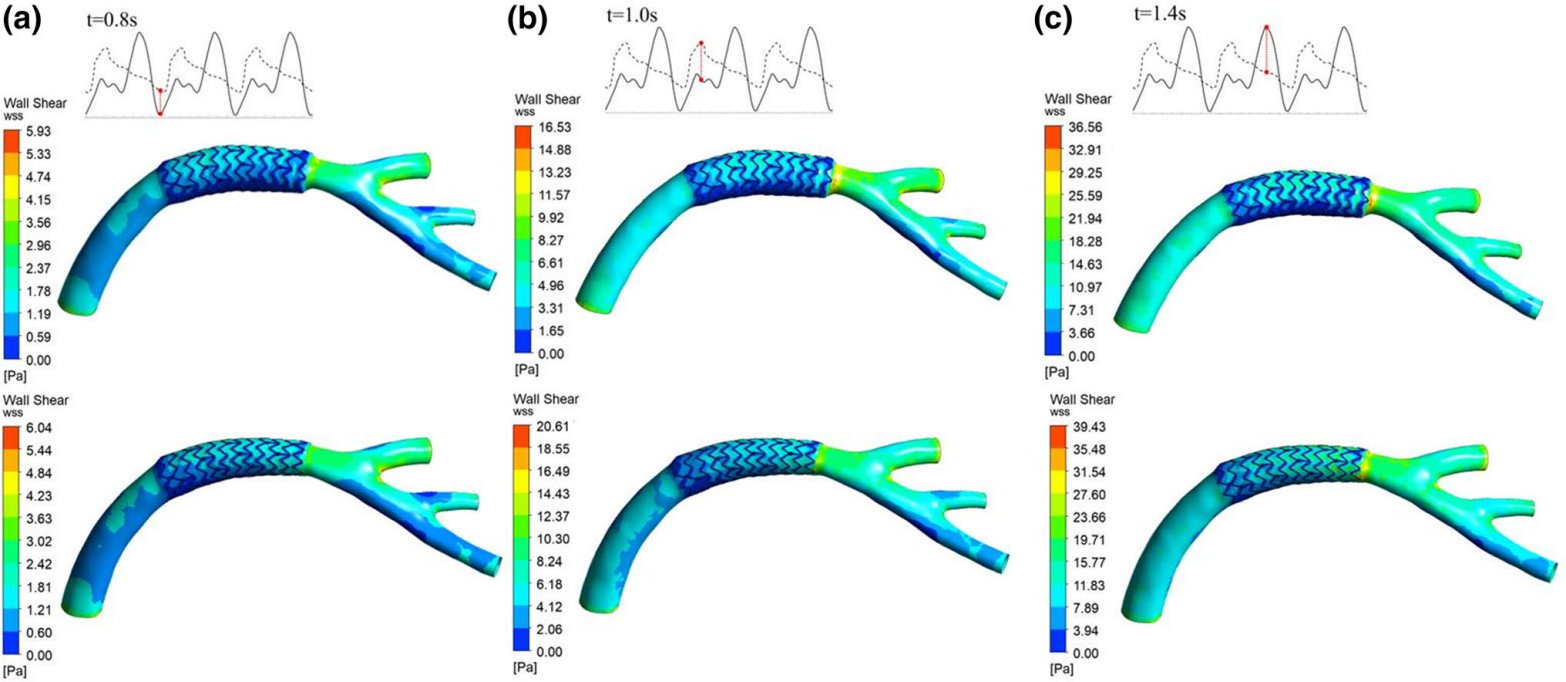
The wall shear stress distribution of the curved coronary vessels in equal diameter expansion and conical expansion at specific time points: **a** 0.8 s, **b** 1.0 s and **c** 1.4 s

The fluid analysis results showed that the maximum wall shear stress appeared at 1.4 s regardless of the implantation mode. For branch coronary vessels, after both equal diameter expansion mode and conical expansion mode stent implantation, the maximum shear stress of the vascular wall at the end reached 75.21 Pa and 72.45 Pa, respectively. For curved coronary vessels, after equal diameter expansion and conical expansion stent implantation, the maximum shear stress of the vascular wall at the end reached 39.43 Pa and 36.56 Pa, respectively. A comparison of the results showed that in different vascular models, the wall shear stress of blood vessels under conical expansion was less than that under equal diameter expansion. Because of the different stent implantation forms, the distribution of vascular wall shear stress is also different. Except for the end area of the blood vessel, the equal diameter expansion mode increases the local wall shear stress. The equal diameter expansion mode causes the local area with large blood vessel deformation at the stent implantation site to be at risk of damage. The distribution of shear stress on the wall of the implanted stent in the conical expansion mode is more uniform than that in the equal diameter expansion mode. There are fewer areas of low shear stress at the end of the stent, and the low shear stress in these areas increases the probability of endothelial hyperplasia that can be seen from the results. Figure 10 shows the wall shear stress distribution of the branch coronary vessels in equal diameter expansion and conical expansion at specific time points. Figure 11 shows the wall shear stress distribution of the curved coronary vessels in equal diameter expansion and conical expansion at specific time points.

## Discussion

Coronary stents, especially metal coronary stents, have been the subject of various research and development studies for more than 30 years (Serruys et al. 1994). Academic and clinical researchers have studied stent indications, stent structure compliance, biological characteristics, and fatigue performance, in addition to a more recent focus on degradable coronary stents (Kumar and Bhatnagar 2021; Peeters et al. 2005). The human coronary artery is not equal in diameter, and its anatomical characteristics show a significant taper (Chiastra et al. 2016; Morlacchi et al. 2013); despite this, current stent products have equal diameter expansion. Therefore, this paper studies the effects of equal diameter expansion mode and conical expansion mode on blood vessels and related hydrodynamic behaviors.

In this study, we found that the conical expansion of the stent has a noticeable impact on the stent and blood vessels (in terms of mechanical behaviors). Compared with the equal diameter expansion, the conical expansion can reduce the stress of the stent and blood vessels. The distal end of the coronary artery is smaller than that of the proximal end. Stent expansion significantly impacts the blood vessels, especially equal diameter expansion, resulting in large deformation of the distal blood vessels, which seriously hampers the performance of the distal blood vessels. This study found that for both equal diameter expansion and conical expansion, the support stress was within a reasonable range in the whole process, and the stress did not exceed the tensile limit of the material. However, the performance of vessel stress in the two situations is different. The effect of equal diameter expansion mode is much greater than conical expansion, which is more evident in the volume of the high-stress area. To reduce the stress at the distal end of the blood vessel, a more feasible approach is to improve the stent structure and associated expansion methods.

Conical expansion coronary stent products are rarely utilized in clinical practice. Although the coronary stent has been used for a long time, research and development focused on conical expansion coronary stent products has been challenging. The main difficulties include: (a) the stent structure design of the conical coronary stent product (He et al. 2020; Khan et al. 2017), which should not only ensure the fatigue performance of the stent but also easily achieve conical expansion; (b) designing an appropriate production process that can follow the current methods used for coronary stent production (with in-depth research being done on various aspects of pressure crimp and anti-unloading); and (c) increasing the amount of research and development focused on conical expansion balloon products, and appropriate matching between the balloon and stent. There are some difficulties associated with the development and marketing of conical expanded coronary artery stent products. Like any vascular stent product, the technology needs to be thoroughly assessed (Wiesent et al. 2019) to verify that the conical expanded coronary artery stent products meet the standards for listing as medical devices. Some companies are currently assessing these novel products and look forward to listing them in the near future.

Although this paper has summarized previous research and presented some new results, it still has its limitations. These limitations include: (a) the lack of real human coronary artery models in the study, and the fact that stent implantation research is relatively similar. Therefore, more human coronary artery models should be obtained to verify the research conclusions of this paper. (b) The lack of a variety of vascular models in the study, including atherosclerotic coronary vessels, which are of clinical significance. (c) The lack of consideration of the fluid–structure coupling between blood vessels and stents. If the fluid–structure coupling analysis between stents and blood vessels can be carried out, it will strengthen the results of this paper. In future research, we will carry out more in-depth studies to address the limitations of the current research and include factors that may influence the clinical deployment of the stents.

## Conclusion

This paper provides a structural-mechanical analysis and hemodynamics assessment of coronary stents implanted into real human coronary vessels. By focusing on the coronary vessels in different parts of the human body, the effects of equal diameter expansion mode and conical expansion mode stents on the mechanical and hemodynamic behaviors of coronary vessels and stents were studied. In addition, the effect of branch vessel fenestration on branch blood flow was also studied. The results of the structural-mechanics analysis showed that different expansion forms of stents had little effect on the mechanical behaviors of stents. However, the effects of equal diameter expansion mode and conical expansion mode on the mechanical behaviors of coronary vessels were different. With the decrease in vessel diameter, the influence of equal diameter expansion mode on the vessel wall is higher than that of conical expansion. The conical expansion makes the stent fit better within the vessel, and the stress is more uniform and will not cause large deformation of the distal vessel. The results of the hemodynamic analysis showed that after stent implantation, the velocity distribution of the six cases was close, and the intravascular blood flow velocity of conical expansion was slightly higher than that of the equal diameter expansion mode. The vortex phenomenon at the end of the vessel caused by equal diameter expansion mode is more evident than conical expansion. This is caused by a large deformation of the vessel’s tail caused by the equal diameter expansion mode of the stent. The existence of an eddy current will increase the adhesion and aggregation of blood cells and make the substances in the blood stay in the eddy current area for a long time. When the stent is implanted at the branch of the blood vessel, the structure of the stent wave rod affects the normal flow of blood into the branch blood vessel. The influence of the stent wave rod on the branch blood vessel can be solved by opening the window of the stent. Wall shear stress analysis of two different coronary vessels showed that the wall shear stress of the equal diameter expansion was greater than that of the conical expansion mode. In the stent implantation area, the wall shear stress of equal diameter expansion mode gradually increases from proximal to distal, resulting in a local area injury risk for the coronary artery. The wall shear stress distribution for conical stent expansion is more uniform compared with equal diameter expansion mode. In the conical coronary artery, the conical expansion mode of the stent slightly improves the blood flow velocity, stabilizes the distribution of wall shear stress generated by expansion, and improves the hydrodynamic environment. All these factors combined can reduce the vascular injury risk and improve the likelihood for the clinical use of a stent.
